# Human Coronaviruses: Insights into Environmental Resistance and Its Influence on the Development of New Antiseptic Strategies

**DOI:** 10.3390/v4113044

**Published:** 2012-11-12

**Authors:** Chloé Geller, Mihayl Varbanov, Raphaël E. Duval

**Affiliations:** UMR 7565, SRSMC, Université de Lorraine – CNRS, Faculty of Pharmacy, 5 rue Albert Lebrun, BP 80403, 54001 Nancy Cedex, FRANCE; Email: chloe.geller@univ-lorraine.fr (C.G.); mihayl.varbanov@univ-lorraine.fr (M.V.)

**Keywords:** human coronaviruses, environmental survival, antiseptics-disinfectants

## Abstract

The *Coronaviridae* family, an enveloped RNA virus family, and, more particularly, human coronaviruses (HCoV), were historically known to be responsible for a large portion of common colds and other upper respiratory tract infections. HCoV are now known to be involved in more serious respiratory diseases, i.e. bronchitis, bronchiolitis or pneumonia, especially in young children and neonates, elderly people and immunosuppressed patients. They have also been involved in nosocomial viral infections. In 2002–2003, the outbreak of severe acute respiratory syndrome (SARS), due to a newly discovered coronavirus, the SARS-associated coronavirus (SARS-CoV); led to a new awareness of the medical importance of the *Coronaviridae* family. This pathogen, responsible for an emerging disease in humans, with high risk of fatal outcome; underline the pressing need for new approaches to the management of the infection, and primarily to its prevention. Another interesting feature of coronaviruses is their potential environmental resistance, despite the accepted fragility of enveloped viruses. Indeed, several studies have described the ability of HCoVs (i.e. HCoV 229E, HCoV OC43 (also known as betacoronavirus 1), NL63, HKU1 or SARS-CoV) to survive in different environmental conditions (e.g. temperature and humidity), on different supports found in hospital settings such as aluminum, sterile sponges or latex surgical gloves or in biological fluids. Finally, taking into account the persisting lack of specific antiviral treatments (there is, in fact, no specific treatment available to fight coronaviruses infections), the *Coronaviridae* specificities (i.e. pathogenicity, potential environmental resistance) make them a challenging model for the development of efficient means of prevention, as an adapted antisepsis-disinfection, to prevent the environmental spread of such infective agents. This review will summarize current knowledge on the capacity of human coronaviruses to survive in the environment and the efficacy of well-known antiseptic-disinfectants against them, with particular focus on the development of new methodologies to evaluate the activity of new antiseptic-disinfectants on viruses.

## 1. Introduction

The worldwide epidemic of SARS (Severe Acute Respiratory Syndrome) in 2002–2003, due to a newly discovered coronavirus, the SARS-CoV (SARS-associated coronavirus), reinforced the interest into the *Coronaviridae* family. Human coronaviruses 229E and OC43 (HCoV 229E and OC43) were previously already known to be responsible for mild and upper respiratory tract diseases. Since then, two further members of this family have been identified (HCoV HUK1 and NL63) and HCoVs have been involved in more serious respiratory tract infections. Moreover, these viruses show an environmental resistance that increases their probability of transfer between contaminated hosts *via* surfaces, hands, *etc*. This resistance leads to the urgent need for development of efficient and targeted modes of prevention. As no treatment or vaccines are available to cure HCoVs infections, it is fundamental to dispose of adapted antiseptics-disinfectants, whose efficiency should be rigorously evaluated.

## 2. Epidemiology and Impact of Coronaviruses in Human Health

### 2.1. Human Coronaviruses Except SARS-CoV

#### 2.1.1. Respiratory Diseases

The HCoV 229E and the HCoV OC43, now called betacoronavirus 1 [1], were the first human coronaviruses to be identified. Since the late sixties, they were recognized as being responsible for upper and mild respiratory tract infections such as the common cold [2,3,4,5,6].

Following the identification of new members of coronaviruses that infect humans, the NL63 in 2004 [7,8,9] and the HKU1 in 2005 [10] and, of course, the SARS-CoV in 2003 [11,12,13,14], new studies have been conducted on the clinical features of HCoVs infections. Indeed, before 2003, very few studies and routine monitoring dealt with the role of coronaviruses in humans. Thus, epidemiological data were rare and it is likely that, as a result, the precise role that HCoVs played in respiratory tract infections was greatly underestimated.

It is important to note that these viruses have been identified worldwide [15,16,17,18,19,20,21,22]. Human coronavirus infections occur mainly in winter, with a short incubation time [19,23,24]. They are recovered in 3 to 11% of patients sampled with a respiratory tract infection, depending on the studied population and the HCoV strain [19,21,23,24,25]. Coronaviruses occupy the fourth or fifth place, behind influenzaviruses, respiratory syncytial virus, adenoviruses and rhinoviruses and their proportion is generally equivalent to the ones of metapneumovirus and parainfluenzaviruses [23,24].

They have since been implicated in more serious diseases of the lower respiratory tract as bronchitis, bronchiolitis or pneumonia [10,26,27,28,29,30,31] or croup in the case of the HCoV NL63 [18,30]. These infections concern predominantly weak patients such as newborns or infants [23,24,26,30,32,33], elderly people [34,35] or immunosuppressed patients [23,36,37]. They have also been implicated in nosocomial infections notably in neonatal care unit [32,33].

#### 2.1.2. Involvement of Coronaviruses in Other Human Diseases

HCoVs are suspected to cause digestive dysfunctions. First, they have been associated with necrotizing enterocolitis in newborns [38], and diarrhea or other gastrointestinal symptoms have been shown to accompany coronavirus infections [17,24,27,30,39]. Then, other findings such as the detection of viral particles and coronavirus RNA in stool samples [39,40], or the presence of HCoV OC43 antibodies in children with gastroenteritis, support this idea. However, despite these arguments, their implication in human intestinal infections is still controversial but should be considered to evaluate the potential routes of HCoVs spread.

Another debate is the potential involvement of HCoVs in central nervous system diseases such as multiple sclerosis. This is supported by a body of evidence, e.g. neurological symptoms in some HCoV OC43 infected patients [29], experimental infection of neural cells with HCoV 229E and OC43 [41,42,43], detection of HCoV 229E and OC43 RNAs and antigens in brain of multiple sclerosis patients [44,45,46], or, more recently, neuroinvasive properties of HCoV OC43 after intranasal inoculation in mice [47]. However, the precise and real implication of HCoVs in neural diseases has not yet been clearly demonstrated.

Furthermore, some studies reported also some heart troubles associated with HCoVs infections [29,48].

### 2.2. A Highly Pathogenic Coronavirus: the SARS-Associated Coronavirus

The epidemic outbreak due to the SARS-CoV was the first worldwide epidemic of the 21st century. It began in Guangdong province of China in November 2002 and spread all over the world within just a few months. This new coronavirus was quickly identified thanks to a concerted international effort [12,13,14,49,50]. 

From November 2002 to July 2003, SARS-CoV affected more than 8000 people in all five continents and caused about 800 deaths [51]. One of the striking features of this epidemic was its nosocomial propagation and the heavy burden of the health care workers [49,52,53,54]. Moreover, the mortality rate was higher than 50% in aged (>60-year-old) populations [55,56,57].

SARS-CoV infection in humans typically causes an influenza-like syndrome such as malaise, rigors, tiredness and high fevers. In one-third of the infected patients, the clinical symptoms regress and patients recover, with, for some of them, persistent pulmonary lesions. In the remaining two-thirds of the infected patients, the disease progresses to an atypical pneumonia. Respiratory insufficiency leading to respiratory failure is the most common cause of death among those infected with SARS-CoV [52,54,58,59]. Many of these patients also develop watery diarrhea with active virus shedding (until several weeks), which might increase the transmissibility of the virus and add another evidence of gastrointestinal tropism of HCoVs [57]. Moreover, the SARS-CoV receptor, the angiotensin-converting enzyme 2 ACE-2, is present in lungs but also in the gastrointestinal tract [60,61].

SARS-CoV seemed predominantly transmitted by respiratory droplets over a relatively close distance [62]. However, direct and indirect contact with respiratory secretions, feces or animal vectors could also lead to transmission, at least under some circumstances [59,63]. 

### 2.3. Evolutionary Ability of Coronaviruses

Besides these pathogenic properties, coronaviruses represent another risk for human population through their interspecies jumping capacity. This is suspected for the HCoV OC43 that may have evolved from the bovine coronavirus, which is responsible for gastrointestinal infections in cattle [64]. Similarly, the SARS-CoV is a zoonotic virus that crossed the species barrier. Phylogenetic analysis of SARS-CoV isolates from animals and humans strongly suggest that the virus originated from animals, most likely bats [65,66,67,68], was amplified in palm civets, and transmitted to human population *via* live animal markets [69].

This potency of coronaviruses may be responsible for new disastrous outbreaks and therefore should be kept in mind.

### 2.4. Vaccines and Therapy

No treatment or vaccine is available to fight HCoVs infections. In the case of SARS-CoV, various approaches were used during the epidemic, but none was really successful and targeted. Treatment was essentially empiric and symptomatic and depended upon the severity of the illness. 

Since then, studies have been conducted to identify potent anti-SARS-CoV treatment. Standard molecules used in viral infections such as ribavirine, interferon or hydrocortisone, were used, leading to diverging, and not so conclusive, results as they were tested *in vivo* or *in vitro* [57,70,71,72,73]. Development of strategies with monoclonal antibodies, siRNAs or molecules such as glycyrrhizin or nelfinavir, have been conducted *in vitro* but still need to be improved [71,74,75,76].

The emergence of the SARS-CoV has also led to the development of new vaccine strategies, including expression of SARS-CoV spike protein in other viruses [77,78,79,80,81,82,83,84,85], inactivated SARS-CoV particles [82,86,87,88,89,90,91] or DNA vaccines [92,93,94,95]. However, an early concern for application of a SARS-CoV vaccine was the experience with animal coronavirus vaccines, which induced enhanced disease and immunopathology in animals when challenged with infectious virus [96]. Indeed, a similar immunopathologic reaction has been described in mice vaccinated with a SARS-CoV vaccine and subsequently challenged with SARS-CoV [97,98,99,100,101]. Thus, safety concerns related to effectiveness and safety for vaccinated persons, especially if exposed to other coronaviruses, should be carefully examined.

## 3. HCoVs: Enveloped, but not that Fragile

In this section, we highlight the potency of coronaviruses to survive in different conditions, despite their enveloped nature. This knowledge is essential for a better understanding of the possibility of virus transfer and cross-contamination, and for formulating appropriate infection-control measures. Indeed, despite the fact that transmission was believed to be mainly achieved by direct physical contact with infected patient or by respiratory droplets, several well-described clusters of infection were difﬁcult to explain by these routes. Examples include transmission to 22 persons on an aircraft [102], to 13 guests sharing the same ﬂoor of a hotel, and more than 300 persons in an apartment complex [103]. These observations led to some speculations about a possible transmission by other means including surfaces, hands, *etc*., and to the study of SARS-CoV (and other HCoVs) survival in different conditions.

Despite the fact that this review is devoted to human coronaviruses, some data concerning the murine hepatitis virus (MHV) and the transmissible gastroenteritis virus (TGEV), now called alphacoronavirus 1 [1], are recorded here because they have been used as SARS-CoV surrogates.

### 3.1. Survival Under Different Conditions of Humidity and Temperature

Some decades ago, a study compared the survival rates of the HCoV 229E to the ones of a non-enveloped virus, the type 1-poliovirus, under different conditions of temperature and humidity. Results are reported in Table 1.

**Table 1:** Survival rates of the HCoV 229E and the poliovirus, type 1, under different conditions of temperature and humidity [104].

	*HCoV 229E*						*Type 1-Poliovirus*,* Sabin strain*			
*Relative humidity*	*20 °C*				*6 °C*		*20 °C*		*6 °C*	
	*15 min*	*24 h*	*72 h*	*6 days*	*15 min*	*24 h*	*15 min*	*24 h*	*15 min*	*24 h*
*30%*	***87%***	***65%***	***>50%***	*n.d.*	*91%*	*65%*	***0%***	***0%***	*n.d.*	*n.d.*
*50%*	***90.9%***	***75%***	***>50% ***	***20%***	*96.5%*	*80%*	***0%***	***0%***	*n.d.*	*n.d.*
*80%*	*55%*	*3%*	*0%*	*n.d.*	***104.8%***	***86%***	*90%*	*30%*	*n.d.*	*n.d.*

(n.d.: not done)

Thus, at 20 °C, aerosolized HCoV 229E was found to better survive at 50% relative humidity than at 30%. Indeed, nearly 20% of the original infectious virus was still detectable after six days. High relative humidity seemed less favorable to the virus, unless the temperature came down to 6 °C. At this temperature, the survival of the HCoV 229E was significantly enhanced whatever the rate of relative humidity. This enhanced survival rate at high relative humidity and low temperature may explain the winter propagation of coronaviruses. Moreover, the HCoV 229E survival was significantly higher at 30% and 50% of relative humidity than those of the poliovirus in the same experimental conditions, which could be a striking result according to its non-enveloped nature [104].

Sensitivity of SARS-CoV to temperature has also been assayed. The exposure of the virus to a temperature of 56 °C over 30 min reduced virus titer under an undetectable level, except if SARS-CoV is associated with proteins, such as 20% fetal calf serum (FCS), which bring a protection for the virus. In this case, the temperature needs to reach 60 °C over 30 min to bring virus titer below the detection limit. This emphasizes the importance of organic material in which viruses could be embedded in the real conditions and could protect the virus, mostly from disinfection procedures. When the virus was placed at 4 °C, there was no loss of infectivity [105]. Another study confirms the viral stability at 4 °C, and also at 20 °C and 37 °C for at least 2 hrs, but SARS-CoV lost its infectivity after 90, 60 and 30 min exposure at 56 °C, 67 °C and 75 °C, respectively [106].

### 3.2. Suspension vs. Desiccation

Coronaviruses also well survive in suspension. At 37 °C, HCoV 229E and OC43 displayed survival rates of 80% and 100%, respectively, in phosphate buffered saline (PBS) over three days and of 30% and 55%, respectively, over six days. These survival rates came down to 50% for HCoV 229E and 30% for HCoV OC43 after three days in culture medium and after ten days, they were of 0% and 10% for each virus, respectively. The same study also showed that desiccation has a more severe effect on coronaviruses. Indeed, in standard environmental conditions (21 °C and 50% to 70% of relative humidity), HCoV 229E infectivity came down to 30% after three hrs of desiccation on various surfaces that can be found in hospital settings, such as aluminum, sterile sponges or surgical latex gloves. HCoV OC43 was more sensitive to desiccation, since its infectivity was below the detectable threshold after three hrs of drying [107].

Rabenau *et al*. made a comparative study on the stability of different viruses, i.e. SARS-CoV, HCoV 229E, type 1-herpes simplex virus (HSV-1) and the type 3-adenovirus, in suspension and after drying. In medium culture, with and without 10% FCS, the HCoV 229E progressively lost its infectivity over nine days, which is consistent with the previous study. The infectious titers of the three other viruses, including the SARS-CoV, were stable over nine days, with and without proteins. After drying on a plastic surface, the HCoV 229E and the HSV-1 lost their infectivity in 72 hrs, in the presence or absence of FCS. In contrast, the SARS-CoV retained its infectivity for as long as six days, with a further protecting effect of proteins. It took nine days in a dried state, for SARS-CoV to completely lose its infectivity. The adenovirus was the most stable virus assayed as it conserved its infectivity throughout the nine days of the experiment [105].

Some other studies confirm these results. SARS-CoV has been shown to survive after drying on different kinds of materials or diluted in water, revealing a decreased infectivity only after 72 to 96 hrs, depending on the conditions. However, its infectivity is reduced more rapidly if it is deposited on porous surfaces such as cotton or paper [106,108].

Thus, RNA of SARS-CoV was found on different environment samples, such as chair, elevator, computer mouse, *etc*., and this may have contributed to contamination of health-care workers who had not been in direct contact with SARS-patients [109,110].

A more recent study implicated water and sewage in the transmission of SARS-CoV, taking the MHV and the TGEV as surrogates for their experiments. At 25 °C, the time required for 99% reduction in water was 22 days for TGEV and 17 days for MHV, and, in sewage, it took nine days for TGEV and seven days for MHV. After four weeks in almost the same conditions but at 4 °C, there was less than <1 log_10_ infectivity decrease for both viruses. The authors concluded that in case of SARS-CoV re-emergence water contaminated with fecal waste should be considered as a potential vehicle of transmission [111].

These studies firmly illustrated the potency of coronaviruses and especially the SARS-CoV, to be transmitted via other routes than respiratory droplets and the likely risk of contamination via surfaces and fomites. It should also be noticed that the residual infectivity of those enveloped viruses in different conditions can almost reach the one of non-enveloped viruses. This reappraises the environmental stability of these two types of viruses.

### 3.3. Influence of pH Conditions on Coronaviruses Survival

The sensitivity of coronaviruses to pH variations has been established for a number of them. They are more stable at slightly acidic pH (6 – 6.5) than at alkaline pH (8). This has been shown for the HCoV 229E [112], the MHV [113,114], the TGEV [115] and the canine coronavirus [116].

### 3.4. Survival in Biological Fluids

As it has been noted earlier, HCoVs are excreted in respiratory secretions but also in other biological fluids such as feces. Knowing and understanding viral survival is then essential to estimate the risk of potential transmission through this route.

Studies have been conducted on SARS-CoV, which was shown to survive at least 96 hrs in sputum, serum and feces. Its infectivity level is nevertheless lower when it is suspended in urines [106]. It is noteworthy that SARS-CoV survival depends on the kind of feces whose pH may vary. Some studies have shown certain surprising results in regard of the previously quoted studies. Indeed, SARS-CoV did not survive beyond 24 hrs in normal feces of an adult or beyond three hrs in newborns’ feces, which is slightly acidic. In contrast, it could survive longer, up to four days, in diarrheic feces whose pH could reach pH 9. The same study revealed a SARS-CoV survival until four to five days in respiratory specimen [108,117].

According to these data, transfer of viruses and cross-contamination should be carefully considered. Indeed, under certain circumstances, for instance in health-care settings, contamination of inanimate materials or other people by infectious respiratory secretions or other body fluids (saliva, urine or feces) seems to play a role in SARS-CoV transmission, and it is likely the same for the other HCoVs. Thus, it is essential to dispose of adapted, targeted and efficient ways of disinfection whose efficiency has to be correctly evaluated.

## 4. Antisepsis-Disinfection: An Efficient Weapon, with Room for Improvement

### 4.1. How Prevention Measures Halted the Propagation of SARS-CoV

The absence of treatment, the high mortality rate and the transmission patterns of SARS-CoV involved the setting of powerful and coordinated means of prevention to stop the worldwide spread of this virus. Indeed, the SARS-CoV epidemic has been brought under control thanks to basic public health measures, including rapid case detection and isolation, contact tracing, quarantine and good precautionary control measures (hand washing, use of personal protective equipment) [54,59]. Additionally, the WHO expressed recommendations for travelers coming from areas affected by the SARS with screening of potential cases and in-flight care of suspected cases followed by aircraft disinfection [121].

Thus, besides these standard measures, our knowledge on HCoVs sensitivity to antiseptics-disinfectants should improve, in order to use these fundamental prevention tools in a targeted and coherent manner.

### 4.2 What is Antisepsis-Disinfection and how do we Evaluate its Efficiency?

Facing the lack in a speciﬁc antiviral treatment, it is necessary to develop new means of prevention and to ensure that the existing ones are efficient according to the field situation. Proper evaluation of the efficiency of antiseptics-disinfectants on viruses is thus crucial. 

Essentially, antiseptic-disinfectant antiviral activity is evaluated by combining viruses and the product to be tested for an appropriately deﬁned and precise contact time, according to the expected use of the product (surface or hands disinfection, for instance). Product activity and its eventual cytotoxicity are then neutralized and the loss of viral infectivity due to the product activity is estimated. Neutralization of the antiseptic-disinfectant activity plays a key role in the test procedure; it ensures a precise contact time, the elimination of the residual activity and cytotoxicity of the tested product, and the successful recovery of viruses that are not killed by the product. These tests require appropriate controls, especially to check the absence of interference on viral infectivity, due to the test itself. It is also important to test the efﬁciency of neutralization, removal of cytotoxicity under reproducible and well-deﬁned test conditions (e.g., contact time and environmental temperature). A germicide can be considered to have an efﬁcient antiseptic-disinfectant antiviral activity if it induces, in a well-deﬁned contact time, a reduction in viral titers higher than 3 or 4 log_10_, depending on American and European regulatory agencies, respectively [122,123]. 

### 4.3. Critical Parameters in Antiviral Antiseptic-Disinfectant Efficiency Evaluation

Some parameters have to be checked particularly carefully to ensure the validity of the results. 

#### 4.3.1. Neutralization Step and Contact Time

The neutralization step plays a key role in this methodology. Several different methods exist to achieve neutralization. The first one is the neutralization by dilution. Theoretically, it allows an instantaneous arrest of the activity of the tested product and the elimination of its cytotoxicity. However, it requires viruses with very high titers in order to observe a reduction in viral titers afterwards. In that case, it is frequently observed that the cytotoxicity is not eliminated thoroughly, making impossible the titration of the virus. Two other techniques are available to counter these drawbacks. Chemical neutralization associates dilution and chemical inactivation of the tested product and its cytotoxicity. However, few neutralizers are available, especially when taking into account the huge number of antiseptics-disinfectants. The gel filtration method allows the retention of antiseptic-disinfectant molecules (and so, their antiviral activity and their cytotoxicity), and the release of viral particles, which could then be tittered. Yet, this method may lengthen the contact time and lead to an overestimation of the product’s activity. Indeed, a precise contact time is fundamental to respect future use conditions and to reflect the real activity of the product in the field. 

#### 4.3.2. Mimics of Field Conditions

Different factors should be considered in order to represent the future use conditions of the product as closely as possible. Different types of tests exist with different levels of evidence: (i) suspension tests, which are useful to screen molecules efficiency and cytotoxicity, (ii) carrier tests, which allow monitoring of the efficiency of the product after viruses have dried on different kinds of surfaces and (iii) in-field tests, for instance, in hospital settings. These are rarely performed because of cost and standardization problems.

In all these tests, organic material (FCS, feces, albumin, *etc*.) could, even should, be added. Indeed, viruses are normally founded embedded in such material protecting them from the action of antiseptics-disinfectants Moreover, a significant part of antiseptics-disinfectants, such as chlorine derived compounds, are inactivated by organic materials. Finally, viruses are known to aggregate themselves and this might be enhanced by the presence of organic material, making them even more resistant to the action of antiseptics-disinfectants.

### 4.4. International Standardization Context

One of the challenges of antiviral antiseptics-disinfectants testing is the standardization to obtain valuable and comparable results. This is illustrated in the next section, where, even if the results concerning the activity of antiseptics-disinfectants on HCoVs are generally consistent with each other, they are still difficult to compare. It is then extremely important to set standards to test these antiseptic activities.

To date, only one European Standard (NF EN 14476+A1) on virucidal antiseptic-disinfectant activity testing in human medicine has been published [122]. This protocol, from January 2007, speciﬁes the test method and the minimum requirements to establish virucidal activity according to the potential use of the products tested, e.g. disinfection of surfaces and instruments, hygienic hand wash or thermochemical disinfection. Virus strains, temperatures, contact times and interfering substances are speciﬁed for each potential use. According to this standard, a product is considered to have an antiseptic-disinfectant antiviral activity if it induces a loss of infectivity of at least 4 log_10_ in viral titers during an accurate contact time.

In the United States, the principal standard is relatively close to the European one but it specifies an efficacy criterion of 3 log_10_. Several standards have been then published to cover the different field situations such as two standards concerning the evaluation of hygienic hand wash, a standard concerning the evaluation of efficacy of virucidal agents intended for inanimate environmental surfaces and, finally, a specific standard concerning the neutralization step [123,124,125,126,127].

### 4.5. Sensitivity of HCoVs to Antiseptics-Disinfectants

#### 4.5.1. Sensitivity of “Classic” HCoVs (other than SARS-CoV) to Antiseptics-Disinfectants

A study, by Sattar *et al*., evaluated the efficiency of 15 antiseptics-disinfectants of various chemical families on four different viruses: two non-enveloped viruses (type b-coxsackievirus and type 5-adenovirus) and two enveloped viruses (HCoV 229E and type 3-parainfluenzavirus). With this aim in view, viral inocula were suspended in feces or mucin to mimic organic matter and left to dry on stainless-steel disks. The contact time was 1 min and the efficacy criterion was a reduction in viral titers of 3 log_10_. Results are gathered in Table 2.

**Table 2:** Comparison of non-enveloped and enveloped viruses (HCoV 229E, type 3-parainfluenzavirus, type b-coxsackievirus and type 5-adenovirus) sensitivity to different antiseptics-disinfectants formulations, thanks to carrier tests [128]. *The efficiency is validated if the reduction in viral titers after a contact-time of 1 min is ≥ 3 log_10_*.

Tested antiseptics-disinfectants	Concentration (%) - (pH at used concentration)	HCoV 229E	Type 3-parainfluenzavirus	Type B-Coxsackievirus	Type 5-Adenovirus
		Enveloped	Enveloped	Non-enveloped	Non-enveloped
**Halogenous compounds**		****	****	****	****
Sodium hypochlorite	0.01 (8.0)	No	No	No	No
	0.10 (9.4)	**Yes**	**Yes**	No	No
	0.50 (11.0)	**Yes**	**Yes**	**Yes**	**Yes**
Chloramine T	0.01 (7.0)	No	**Yes**	No	No
	0.10 (8.0)	**Yes**	No	No	No
	0.30 (8.0)	**Yes**	**Yes**	**Yes**	**Yes**
Sodium hypochlorite and potassium bromide	0.01 (10.0)	No	No	No	No
	0.05 (11.5)	**Yes**	**Yes**	No	No
	0.10 (12.0)	**Yes**	**Yes**	No	No
Povidone-iodine	10.0 (3.0) (1% available iodine)	**Yes**	**Yes**	No	No
**Ethanol**	70.0 (4.0)	**Yes**	**Yes**	No	**Yes**
**Glutaraldehyde**	2.0 (7.0)	**Yes**	**Yes**	**Yes**	**Yes**
**Quaternary ammonium compounds**		****	****	****	****
*n*-alkyl-dimethylbenzyl chloride	0.04 (6.0)	No	No	No	No
*n*-alkyl-dimethylbenzyl chloride	0.04 (1.0)	**Yes**	**Yes**	**Yes**	**Yes**
+ HCl	7.00				
*n*-alkyl-dimethylbenzyl chloride	0.04 (5.0)	**Yes**	**Yes**	No	**Yes**
+ ethanol	70.0				
*n*-alkyl-dimethylbenzyl chloride	0.04 (11.0)	**Yes**	**Yes**	No	**Yes**
+ sodium metasilicate	0.5				
**Chlorhexidine gluconate**	0.008 (5.0)	No	**Yes**	No	No
+ cetrimide	0.08				
Chlorhexidine gluconate	0.05 (4.5)	**Yes**	**Yes**	No	**Yes**
+ cetrimide	0.50				
+ ethanol	70.0				
**Phenolic compounds**		****	****	****	****
*o*-phenylphenol	0.02 (9.0)	No	No	No	No
+ *o*-benzyl-chlorophenol	0.03				
+ *p*-tert-amylphenol	0.01				
*o*-phenylphenol	0.02 (9.0)	**Yes**	**Yes**	No	No
+ *o*-benzyl-chlorophenol l	0.03				
+ *p*-tert-amylpheno	0.01				
+ SDS	0.60				
*o*-phenylphenol	0.02 (9.0)	**Yes**	**Yes**	No	**Yes**
+ *o*-benzyl-chlorophenol	0.03				
+ *p*-tert-amylphenol	0.01				
+ ethanol	70.0				
Sodium* o*-benzyl-*p*-chlorophenate	0.50 (13.0)	**Yes**	**Yes**	**Yes**	**Yes**
+Sodium dodecyl sulfate	0.60				

This study highlighted the fact that enveloped viruses are more sensitive than non-enveloped viruses to the action of antiseptics-disinfectants, despite sensitivity discrepancies within each group. However, enveloped viruses are not that fragile and they are not inactivated by a number of antiseptics-disinfectants such as quaternary ammoniums compounds or phenolic compounds. The association chlorhexidine and cetrimide, widely used in human medicine, did not seem to be effective on HCoV 229E, except if ethanol is added [128].

A more recent study investigated the action of antiseptics-disinfectants on HCoVs 229E and OC43 with suspension tests and contact times of 5 min. The neutralization step was achieved by dilution in medium culture. The povidone-iodine (0.75% free iodine) caused a 50% reduction in infectivity of both of the viruses, which is not enough to claim a virucidal activity. Moreover, to obtain a 50% reduction in HCoV 229E titers, tenfold increase in concentration of povidone-iodine was required. Some other products (70% ethanol, soap or 5% bleach) were assayed but without success because they interfered with the biological viral titration assay [107].

This also highlights the importance of the neutralization step and the necessity of developing means to eliminate the toxicity of the tested products.

This result was also confirmed on the SARS-CoV by Kariwa *et al*. who tested different formulations of povidone-iodine with suspensions tests and contact times of 1 and 2 min. The neutralization step was achieved chemically by the addition of sodium thiosulfate. All formulations reduced the viral infectivity under the detectable level after 2 min of contact-time. The same result was obtained with 70% ethanol in 1 min [129].

Two other studies conducted in our laboratory concerned the HCoV 229E and its sensitivity to two widely used antiseptics, chlorhexidine and hexamidine, but also to new molecules belonging to the calixarene family [130,131]. In these studies, antiseptic antiviral activities were assayed thanks to suspension tests and the efficacy criterion was a reduction of 4 log_10_, as recommended by the European Standard [122]. A novel methodology of gel filtration for the neutralization step was developed in these studies, using homemade and reproducible Sephadex™ columns.

**Figure 1:**
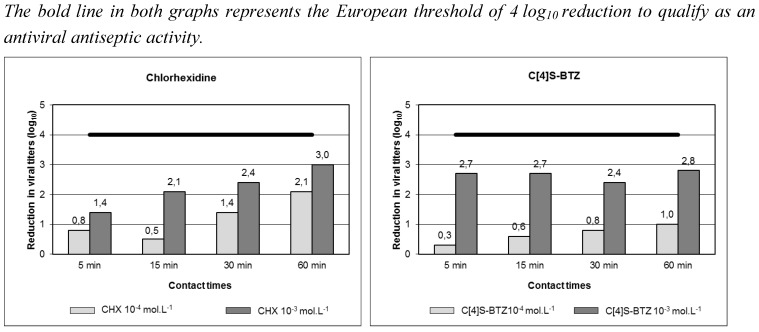
Evaluation of antiseptic HCoV 229E activity of **(a)** chlorhexidine (CHX) and **(b)** the 1,3-bis(bithiazolyl)-tetra-para-sulfonato-calix[4]arene (C[4]S-BTZ) [130,131].

Chlorhexidine was shown to have a time and concentration-dependent anti-HCoV 229E activity allowing a 3 log_10_ reduction, but only after a 60 min contact time (Figure 1a). It was then not sufficient to claim an antiseptic anti-HCoV 229E activity. Hexamidine did not show any activity against HCoV 229E [130,131]. These results highlighted the necessity of (i) evaluating the activity of commonly used antiseptics-disinfectants against different viruses, to be sure of their efficiency and to develop a targeted antisepsis, and (ii) developing new active noncytotoxic molecules.

The second study concerned the antiseptic anti-HCoV 229E activity of two calixarenic compounds, i.e. the tetra-para-sulfonato-calix[4]arene (C[4]S) and the 1,3-bis(bithiazolyl)-tetra-para-sulfonato-calix[4]arene (C[4]S-BTZ) [130,131]. These molecules were attractive targets at first because they did not show any cytotoxicity. Then, the C[4]S-BTZ showed an equivalent, and even better, activity than that of chlorhexidine. Indeed, its activity reached almost 3 log_10_ reduction in viral titers from 5 min of contact time (Figure 1b). Some further studies are needed, but calixarenes appear as interesting candidates to be new antiseptics-disinfectants.

#### 4.5.2. SARS-CoV Sensitivity to Antiseptics-Disinfectants

Rabenau *et al.* achieved a study using suspension tests with different organic loads (albumin, FCS or sheep erythrocytes) and following the recommendations of the European Standard [122]. Most of the tested alcoholic-based solutions (isopropanol or ethanol) has been shown to allow a reduction > 4 log_10_ in viral titers over 30 sec, whatever the added organic load. They also investigated the activity of three surface and instrument disinfectants (one based on benzalkonium chloride and laurylamine; one based on benzalkonium chloride, glutaraldehyde and didecyldimonium chloride; and one based on magnesium monoperphthalate). Contact times were then, still in accordance to the European Standard, 30 and 60 min. SARS-CoV was inactivated by all the disinfectants to below the limit of detection (the smaller reduction factor was 3.25 log_10_), regardless of the type of organic load [132]. The same team pursued its investigation evaluating the SARS-CoV virucidal activity of different disinfectants based on alcohols (propanol, ethanol used for hands disinfection), aldehydes (formaldehyde, glutardialdehyde), glucoprotamin and wine vinegar. The methodology was the same that previously described, except for the organic load, which was FCS. In case of cytotoxic effect after the dilution-neutralization step, the virus-disinfectant mixture was membrane filtered. This allowed the concentration of the viral particles, which could then be tittered, while retaining the disinfectant. The results are recorded in Table 3. The variation in reduction factors was due to the filtration used as neutralization step when disinfectant toxicity was too strong [105].

**Table 3:** Virucidal activity on SARS-CoV of different hand-rub formulations and surfaces disinfectants thanks to suspension tests [105].

Tested formulations	Contact times	Minimal reduction factor (log10)
100% 2-propanol	30 s	≥ 3.31
70% 2-propanol	30 s	≥ 3.31
78% ethanol	30 s	≥ 5.01
45% 2-propanol, 30% 1-propanol	30 s	≥ 2.78
Wine vinegar	60 s	≥ 3.0
0.7% formaldehyde	2 min	≥ 3.01
1.0% formaldehyde	2 min	≥ 3.01
0.5% glutardialdehyde	2 min	≥ 4.01
26% glucoprotamin	2 min	≥ 1.68

Recently, a study used MHV and TGEV as SARS-CoV surrogates. Thanks to carrier tests on stainless steel surfaces and a chemical neutralization step, the anti-SARS-CoV efficacy of six different formulations was evaluated. The efficacy criterion was a reduction of 3 log_10_ in viral titers after 1 min contact time. Results are reported in Table 4.

**Table 4:** Virucidal activity on MHV and TGEV, used as SARS-CoV surrogates, of different hand-rub formulations and surface disinfectants using carrier test methodology [133] *(MHV: Murine hepatitis virus*, *TGEV: Transmissible gastro-enteritis virus).*

Concentration of active ingredients of the tested commercial formulations	MHV	TGEV
Bleach (6% sodium hypochlorite – use dilution: 1:100, ≈ 600 mg/mL)	No	No
9.09% o-phenylphenol, 7.66% p-tertiary amylphenol	No	No
0.55% ortho-phthalaldehyde	No	No
70% ethanol	**Yes**	**Yes**
62% ethanol	No	**Yes**
71% ethanol	No	**Yes**

This study revealed first that there were some behavioral differences between the two viruses chosen as surrogates. This raises the question of the pertinence of surrogates use. However, SARS-CoV is a virus which requires a level 3 containment laboratory. Therefore, virus surrogates allow laboratories, which do not dispose of this type of equipment, to conduct studies and produce precious data without working on a virus, which had already caused a worldwide epidemic.

Another important point revealed by this study is the inefficiency of bleach, a widely used disinfectant, when applied at the 1:100 (0.06%) use-dilution prescribed by the manufacturer. Sattar *et al.*, whose results are recorded in Table 2, have found higher reductions of HCoV 229E viral titers with concentrations of hypochlorite greater than the one tested here. These results are then consistent with a concentration-dependent effect [133].

Another recent study used MHV as the SARS-CoV surrogate, and carrier tests on Petri dishes. Antiseptic antiviral activity of common household disinfectants or antiseptics, containing either 0.05% of triclosan, 0.12% of chloroxylenol, 0.21% of sodium hypochlorite, 0.23% of pine oil, or 0.10% of a quaternary compound with 79.0% of ethanol, were investigated. All of them provided at least a 3 log_10_ reduction in viral titers within a 30 sec contact time, which is consistent with the previous results [134].

Despite the fact that these studies bring vital information, they also highlight the necessity of standardization of the antiseptics-disinfectants activity evaluation. We should also develop in-field tests in order to have a better appreciation of the true action of antiseptics-disinfectants.

## 5. Conclusions

The four HCoVs 229E, OC43, NL63 and HKU1 cause mild respiratory illnesses compared to SARS-CoV, but these infectious agents are involved in 10 to 20% of hospitalizations of young children and immunocompromised adults with respiratory tract illness and they are also involved in nosocomial infections. Moreover, although the SARS-epidemic has been contained, the possibility of re-emergence of SARS-CoV or emergence of another zoonotic strain remains. 

Besides the absence of specific treatment and vaccine, HCoVs are now known to show a significant environmental resistance. Their survival in different biological fluids such as respiratory secretions or feces has been proved. Furthermore, some parameters seem of benefit for HCoVs such as the stabilizing effect of low temperature and high relative humidity or the protective action of organic materials. This protective effect should be carefully considered when developing antiseptic-disinfection strategies. Indeed, this often involves a higher quantity and/or concentration of the antiseptic-disinfectant product and so, a higher toxicity. Thus, an efficient disinfection process should include a precleaning step to get rid of these organic materials. The old well-known principle of antisepsis-disinfection that only clean things can be efficiently disinfected is still valuable.

Finally, in regard to the different studies on HCoVs’s sensitivity to antiseptics-disinfectants, only few formulations are efficient within an adapted contact time and without a too-strong toxicity. For instance, considering their lack of efficiency against HCoVs, and also their toxicity, products only based on quaternary ammoniums or phenolic compounds should be avoided. Some largely used antiseptics-disinfectants such as ethanol or bleach show a significant activity on the HCoVs. However, some critical parameters should be considered, especially in the case of chlorine-derived compounds, such as the presence of organic materials that could prevent their antiseptic activity, or their dose-dependent effect on the HCoVs. The povidone-iodine or the chlorhexidine, when associated to ethanol and/or cetrimide, could be recommended when there is a risk of HCoVs contamination, contrary to another widely used antiseptic, the hexamidine.

It is now essential to pursue investigations on (i) HCoVs’s environmental stability and the role of inanimate material in their spread, (ii) their sensitivity to antiseptics-disinfectants formulations in standardized and targeted conditions, and (iii) the development of new efficient and nontoxic antiseptic-disinfectant molecules such as the calixarenic compounds.

## References

[1] ICTV (International Committee on Taxonomy on Viruses) Virus Taxonomy: 2011 Release (current). http://ictvonline.org/virusTaxonomy.asp?version=2011.

[2] Almeida J.D., Tyrrell D.A. (1967). The morphology of three previously uncharacterized human respiratory viruses that grow in organ culture. J. Gen. Virol..

[3] Bradburne A.F., Bynoe M.L., Tyrrell D.A. (1967). Effects of a "new" human respiratory virus in volunteers. Br. Med. J..

[4] Hamre D., Procknow J.J. (1966). A new virus isolated from the human respiratory tract. Proc. Soc. Exp. Biol. Med..

[5] McIntosh K., Dees J.H., Becker W.B., Kapikian A.Z., Chanock R.M. (1967). Recovery in tracheal organ cultures of novel viruses from patients with respiratory disease. Proc. Natl. Acad. Sci. USA.

[6] Larson H.E., Reed S.E., Tyrrell D.A. (1980). Isolation of rhinoviruses and coronaviruses from 38 colds in adults. J. Med. Virol..

[7] Esper F., Weibel C., Ferguson D., Landry M.L., Kahn J.S. (2005). Evidence of a novel human coronavirus that is associated with respiratory tract disease in infants and young children. J. Infect. Dis..

[8] Fouchier R.A., Hartwig N.G., Bestebroer T.M., Niemeyer B., de Jong J.C., Simon J.H., Osterhaus A.D. (2004). A previously undescribed coronavirus associated with respiratory disease in humans. Proc. Natl. Acad. Sci. USA.

[9] Van der Hoek L., Pyrc K., Jebbink M.F., Vermeulen-Oost W., Berkhout R.J., Wolthers K.C., Wertheim-van Dillen P.M., Kaandorp J., Spaargaren J., Berkhout B. (2004). Identification of a new human coronavirus. Nat. Med..

[10] Woo P.C., Lau S.K., Chu C.M., Chan K.H., Tsoi H.W., Huang Y., Wong B.H., Poon R.W., Cai J.J., Luk W.K. (2005). Characterization and complete genome sequence of a novel coronavirus, coronavirus HKU1, from patients with pneumonia. J. Virol..

[11] Drosten C., Gunther S., Preiser W., van der Werf S., Brodt H.R., Becker S., Rabenau H., Panning M., Kolesnikova L., Fouchier R.A. (2003). Identification of a novel coronavirus in patients with severe acute respiratory syndrome. N. Engl. J. Med..

[12] Ksiazek T.G., Erdman D., Goldsmith C.S., Zaki S.R., Peret T., Emery S., Tong S., Urbani C., Comer J.A., Lim W. (2003). A novel coronavirus associated with severe acute respiratory syndrome. N. Engl. J. Med..

[13] Peiris J.S., Lai S.T., Poon L.L., Guan Y., Yam L.Y., Lim W., Nicholls J., Yee W.K., Yan W.W., Cheung M.T. (2003). Coronavirus as a possible cause of severe acute respiratory syndrome. Lancet.

[14] WHO multicentre collaborative network for Severe Acute Respiratory Syndrome diagnosis (2003). A multicentre collaboration to investigate the cause of severe acute respiratory syndrome. Lancet.

[15] Arden K.E., Nissen M.D., Sloots T.P., Mackay I.M. (2005). New human coronavirus, HCoV-NL63, associated with severe lower respiratory tract disease in Australia. J. Med. Virol..

[16] Bastien N., Anderson K., Hart L., Van Caeseele P., Brandt K., Milley D., Hatchette T., Weiss E.C., Li Y. (2005). Human coronavirus NL63 infection in Canada. J. Infect. Dis..

[17] Vabret A., Mourez T., Dina J., van der Hoek L., Gouarin S., Petitjean J., Brouard J., Freymuth F. (2005). Human coronavirus NL63, France. Emerg. Infect. Dis..

[18] Van der Hoek L., Sure K., Ihorst G., Stang A., Pyrc K., Jebbink M.F., Petersen G., Forster J., Berkhout B., Uberla K. (2005). Croup is associated with the novel coronavirus NL63. PLoS Med..

[19] Gerna G., Percivalle E., Sarasini A., Campanini G., Piralla A., Rovida F., Genini E., Marchi A., Baldanti F. (2007). Human respiratory coronavirus HKU1 versus other coronavirus infections in Italian hospitalised patients. J. Clin. Virol..

[20] Sloots T.P., McErlean P., Speicher D.J., Arden K.E., Nissen M.D., Mackay I.M. (2006). Evidence of human coronavirus HKU1 and human bocavirus in Australian children. J. Clin. Virol..

[21] Chiu S.S., Chan K.H., Chu K.W., Kwan S.W., Guan Y., Poon L.L., Peiris J.S. (2005). Human coronavirus NL63 infection and other coronavirus infections in children hospitalized with acute respiratory disease in Hong Kong, China. Clin. Infect. Dis..

[22] Kon M., Watanabe K., Tazawa T., Tamura T., Tsukagoshi H., Noda M., Kimura H., Mizuta K. (2012). Detection of human coronavirus NL63 and OC43 in children with acute respiratory infections in Niigata, Japan, between 2010 and 2011. Jpn. J. Infect. Dis..

[23] Gerna G., Campanini G., Rovida F., Percivalle E., Sarasini A., Marchi A., Baldanti F. (2006). Genetic variability of human coronavirus OC43-, 229E-, and NL63-like strains and their association with lower respiratory tract infections of hospitalized infants and immunocompromised patients. J. Med. Virol..

[24] Esposito S., Bosis S., Niesters H.G., Tremolati E., Begliatti E., Rognoni A., Tagliabue C., Principi N., Osterhaus A.D. (2006). Impact of human coronavirus infections in otherwise healthy children who attended an emergency department. J. Med. Virol..

[25] Vabret A., Dina J., Gouarin S., Petitjean J., Tripey V., Brouard J., Freymuth F. (2008). Human (non-severe acute respiratory syndrome) coronavirus infections in hospitalised children in France. J. Paediatr. Child. Health..

[26] Talbot H.K., Shepherd B.E., Crowe J.E., Griffin M.R., Edwards K.M., Podsiad A.B., Tollefson S.J., Wright P.F., Williams J.V. (2009). The pediatric burden of human coronaviruses evaluated for twenty years. Pediatr. Infect. Dis. J..

[27] Vabret A., Mourez T., Gouarin S., Petitjean J., Freymuth F. (2003). An outbreak of coronavirus OC43 respiratory infection in Normandy, France. Clin. Infect. Dis..

[28] Van Elden L.J., van Loon A.M., van Alphen F., Hendriksen K.A., Hoepelman A.I., van Kraaij M.G., Oosterheert J.J., Schipper P., Schuurman R., Nijhuis M. (2004). Frequent detection of human coronaviruses in clinical specimens from patients with respiratory tract infection by use of a novel real-time reverse-transcriptase polymerase chain reaction. J. Infect. Dis..

[29] Riski H., Hovi T. (1980). Coronavirus infections of man associated with diseases other than the common cold. J. Med. Virol..

[30] Talbot H.K., Crowe J.E., Edwards K.M., Griffin M.R., Zhu Y., Weinberg G.A., Szilagyi P.G., Hall C.B., Podsiad A.B., Iwane M. (2009). Coronavirus infection and hospitalizations for acute respiratory illness in young children. J. Med. Virol..

[31] Woo P.C., Lau S.K., Tsoi H.W., Huang Y., Poon R.W., Chu C.M., Lee R.A., Luk W.K., Wong G.K., Wong B.H. (2005). Clinical and molecular epidemiological features of coronavirus HKU1-associated community-acquired pneumonia. J. Infect. Dis..

[32] Gagneur A., Sizun J., Vallet S., Legr M.C., Picard B., Talbot P.J. (2002). Coronavirus-related nosocomial viral respiratory infections in a neonatal and paediatric intensive care unit: a prospective study. J. Hosp. Infect..

[33] Sizun J., Soupre D., Legrand M.C., Giroux J.D., Rubio S., Cauvin J.M., Chastel C., Alix D., de Parscau L. (1995). Neonatal nosocomial respiratory infection with coronavirus: a prospective study in a neonatal intensive care unit. Acta Paediatr..

[34] Falsey A.R., Walsh E.E., Hayden F.G. (2002). Rhinovirus and coronavirus infection-associated hospitalizations among older adults. J. Infect. Dis..

[35] Nicholson K.G., Kent J., Hammersley V., Cancio E. (1997). Acute viral infections of upper respiratory tract in elderly people living in the community: comparative, prospective, population based study of disease burden. BMJ.

[36] Pene F., Merlat A., Vabret A., Rozenberg F., Buzyn A., Dreyfus F., Cariou A., Freymuth F., Lebon P. (2003). Coronavirus 229E-related pneumonia in immunocompromised patients. Clin. Infect. Dis..

[37] Folz R.J., Elkordy M.A. (1999). Coronavirus pneumonia following autologous bone marrow transplantation for breast cancer. Chest.

[38] Chany C., Moscovici O., Lebon P., Rousset S. (1982). Association of coronavirus infection with neonatal necrotizing enterocolitis. Pediatrics.

[39] Vabret A., Dina J., Gouarin S., Petitjean J., Corbet S., Freymuth F. (2006). Detection of the new human coronavirus HKU1: a report of 6 cases. Clin. Infect. Dis..

[40] Zhang X.M., Herbst W., Kousoulas K.G., Storz J. (1994). Biological and genetic characterization of a hemagglutinating coronavirus isolated from a diarrhoeic child. J. Med. Virol..

[41] Arbour N., Cote G., Lachance C., Tardieu M., Cashman N.R., Talbot P.J. (1999). Acute and persistent infection of human neural cell lines by human coronavirus OC43. J. Virol..

[42] Arbour N., Ekande S., Cote G., Lachance C., Chagnon F., Tardieu M., Cashman N.R., Talbot P.J. (1999). Persistent infection of human oligodendrocytic and neuroglial cell lines by human coronavirus 229E. J. Virol..

[43] Bonavia A., Arbour N., Yong V.W., Talbot P.J. (1997). Infection of primary cultures of human neural cells by human coronaviruses 229E and OC43. J. Virol..

[44] Arbour N., Day R., Newcombe J., Talbot P.J. (2000). Neuroinvasion by human respiratory coronaviruses. J. Virol..

[45] Murray R.S., Brown B., Brian D., Cabirac G.F. (1992). Detection of coronavirus RNA and antigen in multiple sclerosis brain. Ann. Neurol..

[46] Stewart J.N., Mounir S., Talbot P.J. (1992). Human coronavirus gene expression in the brains of multiple sclerosis patients. Virology.

[47] St-Jean J.R., Jacomy H., Desforges M., Vabret A., Freymuth F., Talbot P.J. (2004). Human respiratory coronavirus OC43: genetic stability and neuroinvasion. J. Virol..

[48] Riski H., Hovi T., Frick M.H. (1980). Carditis associated with coronavirus infection. Lancet.

[49] Hsu L.Y., Lee C.C., Green J.A., Ang B., Paton N.I., Lee L., Villacian J.S., Lim P.L., Earnest A., Leo Y.S. (2003). Severe acute respiratory syndrome (SARS) in Singapore: clinical features of index patient and initial contacts. Emerg. Infect. Dis..

[50] Poutanen S.M., Low D.E., Henry B., Finkelstein S., Rose D., Green K., Tellier R., Draker R., Adachi D., Ayers M. (2003). Identification of severe acute respiratory syndrome in Canada. N. Engl. J. Med..

[51] WHO (World Health Organization) Summary of probable SARS cases with onset of illness from 1 November 2002 to 31 July.

[52] Lee N., Hui D., Wu A., Chan P., Cameron P., Joynt G.M., Ahuja A., Yung M.Y., Leung C.B., To K.F. (2003). A major outbreak of severe acute respiratory syndrome in Hong Kong. N. Engl. J. Med..

[53] Varia M., Wilson S., Sarwal S., McGeer A., Gournis E., Galanis E., Henry B. (2003). Investigation of a nosocomial outbreak of severe acute respiratory syndrome (SARS) in Toronto, Canada. CMAJ.

[54] Zhao Z., Zhang F., Xu M., Huang K., Zhong W., Cai W., Yin Z., Huang S., Deng Z., Wei M. (2003). Description and clinical treatment of an early outbreak of severe acute respiratory syndrome (SARS) in Guangzhou, PR China. J. Med. Microbiol..

[55] Booth C.M., Matukas L.M., Tomlinson G.A., Rachlis A.R., Rose D.B., Dwosh H.A., Walmsley S.L., Mazzulli T., Avendano M., Derkach P. (2003). Clinical features and short-term outcomes of 144 patients with SARS in the greater Toronto area. JAMA.

[56] Donnelly C.A., Ghani A.C., Leung G.M., Hedley A.J., Fraser C., Riley S., Abu-Raddad L.J., Ho L.M., Thach T.Q., Chau P. (2003). Epidemiological determinants of spread of causal agent of severe acute respiratory syndrome in Hong Kong. Lancet.

[57] Peiris J.S., Chu C.M., Cheng V.C., Chan K.S., Hung I.F., Poon L.L., Law K.I., Tang B.S., Hon T.Y., Chan C.S. (2003). Clinical progression and viral load in a community outbreak of coronavirus-associated SARS pneumonia: a prospective study. Lancet.

[58] Peiris J.S., Yuen K.Y., Osterhaus A.D., Stohr K. (2003). The severe acute respiratory syndrome. N. Engl. J. Med..

[59] WHO (World Health Organization) Consensus document on the epidemiology of severe acute respiratory syndrome (SARS). http://www.who.int/csr/sars/en/WHOconsensus.pdf.

[60] Hamming I., Timens W., Bulthuis M.L., Lely A.T., Navis G.J., van Goor H. (2004). Tissue distribution of ACE2 protein, the functional receptor for SARS coronavirus. A first step in understanding SARS pathogenesis. J. Pathol..

[61] Li W., Moore M.J., Vasilieva N., Sui J., Wong S.K., Berne M.A., Somasundaran M., Sullivan J.L., Luzuriaga K., Greenough T.C. (2003). Angiotensin-converting enzyme 2 is a functional receptor for the SARS coronavirus. Nature.

[62] Dwosh H.A., Hong H.H., Austgarden D., Herman S., Schabas R. (2003). Identification and containment of an outbreak of SARS in a community hospital. CMAJ.

[63] Ng S.K. (2003). Possible role of an animal vector in the SARS outbreak at Amoy Gardens. Lancet.

[64] Vijgen L., Keyaerts E., Moes E., Thoelen I., Wollants E., Lemey P., Vandamme A.M., Van Ranst M. (2005). Complete genomic sequence of human coronavirus OC43: molecular clock analysis suggests a relatively recent zoonotic coronavirus transmission event. J. Virol..

[65] Lau S.K., Woo P.C., Li K.S., Huang Y., Tsoi H.W., Wong B.H., Wong S.S., Leung S.Y., Chan K.H., Yuen K.Y. (2005). Severe acute respiratory syndrome coronavirus-like virus in Chinese horseshoe bats. Proc. Natl. Acad. Sci. USA.

[66] Li W., Shi Z., Yu M., Ren W., Smith C., Epstein J.H., Wang H., Crameri G., Hu Z., Zhang H. (2005). Bats are natural reservoirs of SARS-like coronaviruses. Science.

[67] Poon L.L., Chu D.K., Chan K.H., Wong O.K., Ellis T.M., Leung Y.H., Lau S.K., Woo P.C., Suen K.Y., Yuen K.Y. (2005). Identification of a novel coronavirus in bats. J. Virol..

[68] Woo P.C., Lau S.K., Li K.S., Poon R.W., Wong B.H., Tsoi H.W., Yip B.C., Huang Y., Chan K.H., Yuen K.Y. (2006). Molecular diversity of coronaviruses in bats. Virology.

[69] Kan B., Wang M., Jing H., Xu H., Jiang X., Yan M., Liang W., Zheng H., Wan K., Liu Q. (2005). Molecular evolution analysis and geographic investigation of severe acute respiratory syndrome coronavirus-like virus in palm civets at an animal market and on farms. J. Virol..

[70] Cinatl J., Michaelis M., Morgenstern B., Doerr H.W. (2005). High-dose hydrocortisone reduces expression of the pro-inflammatory chemokines CXCL8 and CXCL10 in SARS coronavirus-infected intestinal cells. Int. J. Mol. Med..

[71] Cinatl J., Morgenstern B., Bauer G., Chandra P., Rabenau H., Doerr H.W. (2003). Glycyrrhizin, an active component of liquorice roots, and replication of SARS-associated coronavirus. Lancet.

[72] Morgenstern B., Michaelis M., Baer P.C., Doerr H.W., Cinatl J. (2005). Ribavirin and interferon-beta synergistically inhibit SARS-associated coronavirus replication in animal and human cell lines. Biochem. Biophys. Res. Commun..

[73] Mazzulli T., Farcas G.A., Poutanen S.M., Willey B.M., Low D.E., Butany J., Asa S.L., Kain K.C. (2004). Severe acute respiratory syndrome-associated coronavirus in lung tissue. Emerg. Infect. Dis..

[74] Yamamoto N., Yang R., Yoshinaka Y., Amari S., Nakano T., Cinatl J., Rabenau H., Doerr H.W., Hunsmann G., Otaka A. (2004). HIV protease inhibitor nelfinavir inhibits replication of SARS-associated coronavirus. Biochem. Biophys. Res. Commun..

[75] Cinatl J., Michaelis M., Hoever G., Preiser W., Doerr H.W. (2005). Development of antiviral therapy for severe acute respiratory syndrome. Antiviral Res..

[76] Haagmans B.L., Osterhaus A.D. (2006). Coronaviruses and their therapy. Antiviral Res..

[77] Bisht H., Roberts A., Vogel L., Bukreyev A., Collins P.L., Murphy B.R., Subbarao K., Moss B. (2004). Severe acute respiratory syndrome coronavirus spike protein expressed by attenuated vaccinia virus protectively immunizes mice. Proc. Natl. Acad. Sci. USA..

[78] Kapadia S.U., Rose J.K., Lamirande E., Vogel L., Subbarao K., Roberts A. (2005). Long-term protection from SARS coronavirus infection conferred by a single immunization with an attenuated VSV-based vaccine. Virology.

[79] Chen Z., Zhang L., Qin C., Ba L., Yi C.E., Zhang F., Wei Q., He T., Yu W., Yu J. (2005). Recombinant modified vaccinia virus Ankara expressing the spike glycoprotein of severe acute respiratory syndrome coronavirus induces protective neutralizing antibodies primarily targeting the receptor binding region. J. Virol..

[80] Bukreyev A., Lamirande E.W., Buchholz U.J., Vogel L.N., Elkins W.R., St Claire M., Murphy B.R., Subbarao K., Collins P.L. (2004). Mucosal immunisation of African green monkeys (Cercopithecus aethiops) with an attenuated parainfluenza virus expressing the SARS coronavirus spike protein for the prevention of SARS. Lancet.

[81] Ishii K., Hasegawa H., Nagata N., Mizutani T., Morikawa S., Suzuki T., Taguchi F., Tashiro M., Takemori T., Miyamura T. (2006). Induction of protective immunity against severe acute respiratory syndrome coronavirus (SARS-CoV) infection using highly attenuated recombinant vaccinia virus DIs. Virology.

[82] See R.H., Zakhartchouk A.N., Petric M., Lawrence D.J., Mok C.P., Hogan R.J., Rowe T., Zitzow L.A., Karunakaran K.P., Hitt M.M. (2006). Comparative evaluation of two severe acute respiratory syndrome (SARS) vaccine candidates in mice challenged with SARS coronavirus. J. Gen. Virol..

[83] Czub M., Weingartl H., Czub S., He R., Cao J. (2005). Evaluation of modified vaccinia virus Ankara based recombinant SARS vaccine in ferrets. Vaccine.

[84] He Y., Li J., Heck S., Lustigman S., Jiang S. (2006). Antigenic and immunogenic characterization of recombinant baculovirus-expressed severe acute respiratory syndrome coronavirus spike protein: implication for vaccine design. J. Virol..

[85] Zhou Z., Post P., Chubet R., Holtz K., McPherson C., Petric M., Cox M. (2006). A recombinant baculovirus-expressed S glycoprotein vaccine elicits high titers of SARS-associated coronavirus (SARS-CoV) neutralizing antibodies in mice. Vaccine.

[86] He Y., Zhou Y., Siddiqui P., Jiang S. (2004). Inactivated SARS-CoV vaccine elicits high titers of spike protein-specific antibodies that block receptor binding and virus entry. Biochem. Biophys. Res. Commun..

[87] Spruth M., Kistner O., Savidis-Dacho H., Hitter E., Crowe B., Gerencer M., Bruhl P., Grillberger L., Reiter M., Tauer C. (2006). A double-inactivated whole virus candidate SARS coronavirus vaccine stimulates neutralising and protective antibody responses. Vaccine.

[88] Tang L., Zhu Q., Qin E., Yu M., Ding Z., Shi H., Cheng X., Wang C., Chang G., Fang F. (2004). Inactivated SARS-CoV vaccine prepared from whole virus induces a high level of neutralizing antibodies in BALB/c mice. DNA Cell Biol..

[89] Xiong S., Wang Y.F., Zhang M.Y., Liu X.J., Zhang C.H., Liu S.S., Qian C.W., Li J.X., Lu J.H., Wan Z.Y. (2004). Immunogenicity of SARS inactivated vaccine in BALB/c mice. Immunol. Lett..

[90] Takasuka N., Fujii H., Takahashi Y., Kasai M., Morikawa S., Itamura S., Ishii K., Sakaguchi M., Ohnishi K., Ohshima M. (2004). A subcutaneously injected UV-inactivated SARS coronavirus vaccine elicits systemic humoral immunity in mice. Int. Immunology.

[91] Stadler K., Roberts A., Becker S., Vogel L., Eickmann M., Kolesnikova L., Klenk H.D., Murphy B., Rappuoli R., Abrignani S. (2005). SARS vaccine protective in mice. Emerg. Infect. Dis..

[92] Woo P.C., Lau S.K., Tsoi H.W., Chen Z.W., Wong B.H., Zhang L., Chan J.K., Wong L.P., He W., Ma C. (2005). SARS coronavirus spike polypeptide DNA vaccine priming with recombinant spike polypeptide from Escherichia coli as booster induces high titer of neutralizing antibody against SARS coronavirus. Vaccine.

[93] Yang Z.Y., Kong W.P., Huang Y., Roberts A., Murphy B.R., Subbarao K., Nabel G.J. (2004). A DNA vaccine induces SARS coronavirus neutralization and protective immunity in mice. Nature.

[94] Zhao P., Ke J.S., Qin Z.L., Ren H., Zhao L.J., Yu J.G., Gao J., Zhu S.Y., Qi Z.T. (2004). DNA vaccine of SARS-Cov S gene induces antibody response in mice. Acta Biochim. Biophys. Sin..

[95] Wang Z., Yuan Z., Matsumoto M., Hengge U.R., Chang Y.F. (2005). Immune responses with DNA vaccines encoded different gene fragments of severe acute respiratory syndrome coronavirus in BALB/c mice. Biochem. Biophys. Res. Commun..

[96] Perlman S., Dandekar A.A. (2005). Immunopathogenesis of coronavirus infections: implications for SARS. Nat. Rev. Immunol..

[97] Bolles M., Deming D., Long K., Agnihothram S., Whitmore A., Ferris M., Funkhouser W., Gralinski L., Totura A., Heise M. (2011). A double-inactivated severe acute respiratory syndrome coronavirus vaccine provides incomplete protection in mice and induces increased eosinophilic proinflammatory pulmonary response upon challenge. J. Virol..

[98] Deming D., Sheahan T., Heise M., Yount B., Davis N., Sims A., Suthar M., Harkema J., Whitmore A., Pickles R. (2006). Vaccine efficacy in senescent mice challenged with recombinant SARS-CoV bearing epidemic and zoonotic spike variants. PLoS Med..

[99] Lokugamage K.G., Yoshikawa-Iwata N., Ito N., Watts D.M., Wyde P.R., Wang N., Newman P., Kent Tseng C.T., Peters C.J., Makino S. (2008). Chimeric coronavirus-like particles carrying severe acute respiratory syndrome coronavirus (SCoV) S protein protect mice against challenge with SCoV. Vaccine.

[100] Yasui F., Kai C., Kitabatake M., Inoue S., Yoneda M., Yokochi S., Kase R., Sekiguchi S., Morita K., Hishima T. (2008). Prior immunization with severe acute respiratory syndrome (SARS)-associated coronavirus (SARS-CoV) nucleocapsid protein causes severe pneumonia in mice infected with SARS-CoV. J. Immunol..

[101] Tseng C.T., Sbrana E., Iwata-Yoshikawa N., Newman P.C., Garron T., Atmar R.L., Peters C.J., Couch R.B. (2012). Immunization with SARS coronavirus vaccines leads to pulmonary immunopathology on challenge with the SARS virus. PLoS ONE..

[102] Olsen S.J., Chang H.L., Cheung T.Y., Tang A.F., Fisk T.L., Ooi S.P., Kuo H.W., Jiang D.D., Chen K.T., Lando J. (2003). Transmission of the severe acute respiratory syndrome on aircraft. N. Engl. J. Med..

[103] Lee S.H. (2003). The SARS epidemic in Hong Kong. J. Epidemiol. Community Health..

[104] Ijaz M.K., Brunner A.H., Sattar S.A., Nair R.C., Johnson-Lussenburg C.M. (1985). Survival characteristics of airborne human coronavirus 229E. J. Gen. Virol..

[105] Rabenau H.F., Cinatl J., Morgenstern B., Bauer G., Preiser W., Doerr H.W. (2005). Stability and inactivation of SARS coronavirus. Med. Microbiol. Immunol..

[106] Duan S.M., Zhao X.S., Wen R.F., Huang J.J., Pi G.H., Zhang S.X., Han J., Bi S.L., Ruan L., Dong X.P. (2003). Stability of SARS coronavirus in human specimens and environment and its sensitivity to heating and UV irradiation. Biomed. Environ. Sci..

[107] Sizun J., Yu M.W., Talbot P.J. (2000). Survival of human coronaviruses 229E and OC43 in suspension and after drying on surfaces: a possible source of hospital-acquired infections. J. Hosp. Infect..

[108] Lai M.Y., Cheng P.K., Lim W.W. (2005). Survival of severe acute respiratory syndrome coronavirus. Clin. Infect. Dis..

[109] Chen Y.C., Huang L.M., Chan C.C., Su C.P., Chang S.C., Chang Y.Y., Chen M.L., Hung C.C., Chen W.J., Lin F.Y. (2004). SARS in hospital emergency room. Emerg. Infect. Dis..

[110] Dowell S.F., Simmerman J.M., Erdman D.D., Wu J.S., Chaovavanich A., Javadi M., Yang J.Y., Anderson L.J., Tong S., Ho M.S. (2004). Severe acute respiratory syndrome coronavirus on hospital surfaces. Clin. Infect. Dis..

[111] Casanova L., Rutala W.A., Weber D.J., Sobsey M.D. (2009). Survival of surrogate coronaviruses in water. Water Res..

[112] Lamarre A., Talbot P.J. (1989). Effect of pH and temperature on the infectivity of human coronavirus 229E. Can. J. Microbiol..

[113] Sturman L.S., Ricard C.S., Holmes K.V. (1990). Conformational change of the coronavirus peplomer glycoprotein at pH 8.0 and 37 degrees C correlates with virus aggregation and virus-induced cell fusion. J. Virol..

[114] Daniel C., Talbot P.J. (1987). Physico-chemical properties of murine hepatitis virus, strain A 59. Brief report. Arch. Virol..

[115] Pocock D.H., Garwes D.J. (1975). The influence of pH on the growth and stability of transmissible gastroenteritis virus in vitro. Arch. Virol..

[116] Pratelli A. (2008). Canine coronavirus inactivation with physical and chemical agents. Vet. J..

[117] WHO (World Health Organization) First data on stability and resistance of SARS coronavirus compiled by members of WHO laboratory network. http://www.who.int/csr/sars/survival_2003_05_04/en/index.html.

[118] Lipsitch M., Cohen T., Cooper B., Robins J.M., Ma S., James L., Gopalakrishna G., Chew S.K., Tan C.C., Samore M.H. (2003). Transmission dynamics and control of severe acute respiratory syndrome. Science.

[119] Riley S., Fraser C., Donnelly C.A., Ghani A.C., Abu-Raddad L.J., Hedley A.J., Leung G.M., Ho L.M., Lam T.H., Thach T.Q. (2003). Transmission dynamics of the etiological agent of SARS in Hong Kong: impact of public health interventions. Science.

[120] Seto W.H., Tsang D., Yung R.W., Ching T.Y., Ng T.K., Ho M., Ho L.M., Peiris J.S. (2003). Effectiveness of precautions against droplets and contact in prevention of nosocomial transmission of severe acute respiratory syndrome (SARS). Lancet.

[121] WHO (World Health Organization) (2003). Global surveillance for Severe Acute Respiratory Syndrome (SARS). Wkly. Epidemiol. Rec..

[122] AFNOR (2007). Chemical disinfectants and- Virucidal quantitative suspension test for chemical disinfectants and antiseptics used in human medicine - Test method and requirements (phase 2, step 1). NF EN 14476+A1.

[123] ASTM (1997). Standard Test Method for Efficacy of Virucidal Agents Intended for Inanimate Environmental Surfaces. E1053-97 (last reapproval in 2002).

[124] ASTM (1996). Standard Test Method for Efficacy of Antimicrobial Agents Against Viruses in Suspension. E1052-96 (last reapproval in 2002).

[125] ASTM (2004). Standard Test Method for Neutralization of Virucidal Agents in Virucidal Efficacy Evaluations. E1482-04.

[126] ASTM (American Society for Testing and Materials) (2002). Standard Test Method for Determining the Virus-Eliminating Effectiveness of Liquid Hygienic Handwash and Handrub Agents Using the Fingerpads of Adult Volunteers. E1838-02.

[127] ASTM (American Society for Testing and Materials) (2009). Standard Test Method for Evaluation of Hygienic Handwash and Handrub Formulations for Virus-Eliminating Activity Using the Entire Hand. E2011-09.

[128] Sattar S.A., Springthorpe V.S., Karim Y., Loro P. (1989). Chemical disinfection of non-porous inanimate surfaces experimentally contaminated with four human pathogenic viruses. Epidemiol. Infect..

[129] Kariwa H., Fujii N., Takashima I. (2006). Inactivation of SARS coronavirus by means of povidone-iodine, physical conditions and chemical reagents. Dermatology..

[130] Geller C., Fontanay S., Finance C., Duval R.E. (2009). A new Sephadex-based method for removing microbicidal and cytotoxic residues when testing antiseptics against viruses: Experiments with a human coronavirus as a model. J. Virol. Methods..

[131] Geller C., Fontanay S., Mourer M., Dibama H.M., Regnouf-de-Vains J.B., Finance C., Duval R.E. (2010). Antiseptic properties of two calix[4]arenes derivatives on the human coronavirus 229E. Antiviral Res..

[132] Rabenau H.F., Kampf G., Cinatl J., Doerr H.W. (2005). Efficacy of various disinfectants against SARS coronavirus. J. Hosp. Infect..

[133] Hulkower R.L., Casanova L.M., Rutala W.A., Weber D.J., Sobsey M.D. (2011). Inactivation of surrogate coronaviruses on hard surfaces by health care germicides. Am. J. Infect. Control..

[134] Dellanno C., Vega Q., Boesenberg D. (2009). The antiviral action of common household disinfectants and antiseptics against murine hepatitis virus, a potential surrogate for SARS coronavirus. Am. J. Infect. Control..

